# Targeted Social Distancing Designs for Pandemic Influenza

**DOI:** 10.3201/eid1211.060255

**Published:** 2006-11

**Authors:** Robert J. Glass, Laura M. Glass, Walter E. Beyeler, H. Jason Min

**Affiliations:** * Sandia National Laboratories, Albuquerque, New Mexico, USA;; † Albuquerque Public High School, Albuquerque, New Mexico, USA

**Keywords:** Influenza, social networks, social distance, computer simulation, nonlinear models, research

## Abstract

Targeted social distancing to mitigate pandemic influenza can be designed through simulation of influenza's spread within local community social contact networks. We demonstrate this design for a stylized community representative of a small town in the United States. The critical importance of children and teenagers in transmission of influenza is first identified and targeted. For influenza as infectious as 1957–58 Asian flu (≈50% infected), closing schools and keeping children and teenagers at home reduced the attack rate by >90%. For more infectious strains, or transmission that is less focused on the young, adults and the work environment must also be targeted. Tailored to specific communities across the world, such design would yield local defenses against a highly virulent strain in the absence of vaccine and antiviral drugs.

## Abstract 2

Local community networks can mitigate pandemic influenza in the absence of vaccine and antiviral drugs.

At the start of an influenza pandemic, effective vaccine and antiviral drugs may not be available to the general population (*1**,**2*). If the accompanying illness and death rates of the virus strain are high, how might a community respond to protect itself? Closing roads, restricting travel, and community-level quarantine will enter discussions (*3**,**4*). However, within a community, influenza spreads from person to person through the social contact network. Therefore, understanding and strategically controlling this network during a period of pandemic is critical.

We describe how social contact network–focused mitigation can be designed. At the foundation of the design process is a network-based simulation model for the spread of influenza. We apply this model to a community of 10,000 persons connected within an overlapping, stylized, social network representative of a small US town. After study of the unmitigated transmission of influenza within the community, we change the frequency of contact within targeted groups and build combinations of strategies that can contain the epidemic. Finally, we show how infectivity of the strain and underlying structure of the infectious contact network influence the design of social distancing strategies. In the absence of vaccine and antiviral drugs, design for specific communities would defend against highly virulent influenza.

## Methods

The design process first creates an explicit social contact network in which persons are linked to others in a community. Spread of influenza within the network is then simulated by imposing behavioral rules for persons, their links, and the disease. These rules are modified to implement targeted mitigation strategies within the community, the effectiveness of which is evaluated (*5*).

## Contact Network

A network is created by specifying groups of given sizes (or range of sizes) within which persons of specified ages interact (e.g., school classes, households, clubs). The average number of links per person within the group is also specified because cliques form or are imposed (e.g., seating in a classroom). This number is used to construct a within-group network that can take various forms. We used fully connected, random, or ring networks for each group. Random networks are formed by randomly choosing 2 persons within the group and linking them. This process is repeated until the number of links within the group yields the specified average (each person will have a different number of links). The ring is formed by first placing persons next to neighbors and linking them to form a complete circle. Additional links are then made to next nearest neighbors symmetrically around the ring. Finally, links within a group are given an average frequency of contact per day. With this approach, a network can be built from the experience of community members to exhibit the clustered yet small-world characteristics (*6*) and overlapping quality of a structured community (*7**,**8*).

**Figure 1:**
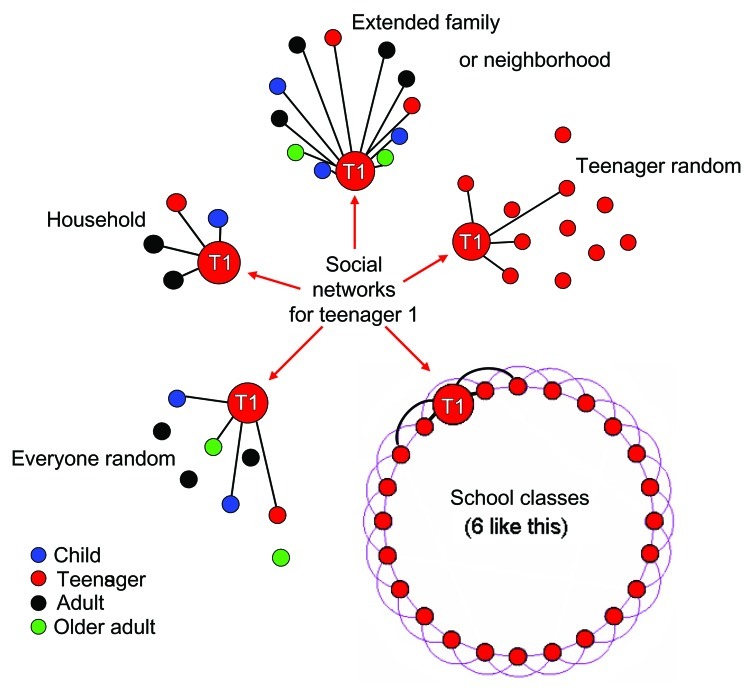
Typical groups and person-to-person links for model teenager. The teenager (T1) belongs to a household (fully connected network, mean link contact frequency 6/day), an extended family or neighborhood (fully connected network, mean link contact frequency 1/day), and 6 school classes (ring network with connections to 2 other teenagers on each side as shown in black; purple links denote connections of other teenagers within the class; mean link contact frequency 1/day). Two random networks are also imposed, 1 within the age group (teenager random, average of 3 links/teenager, mean link contact frequency of 1/day), and 1 across all age groups (overall random average of 25 links/person [not all links shown], mean link contact frequency of 0.04/day).

Our network represented a stylized small US town and took advantage of the diverse backgrounds of the authors (1 of whom is a teenager). The population of 10,000 conformed to the 2000 Census (*9*) and consisted of children (<11 years of age, 17.7%), teenagers (12–18 years of age, 11.3%), adults (19–64 years of age, 58.5%), and older adults (>65 years of age, 12.5%). All persons belonged to multiple groups, each associated with a subnetwork of links that reflected their lives within the community (Figure 1, Table 1). Households were composed of families (adults with children or teenagers), adults, or older adults. The age-class makeup and size of households conformed to the 2000 Census (*9*). All persons within each household were linked to each other with mean link contact frequencies of 6/day. Every person also belonged to 1 multiage extended family (or neighborhood) group (mean membership 12.5, mean link contact frequency 1/day).

**Table 1:** Groups, membership, networks, and mean frequencies of contact per link

Group (no. groups in community)	Membership	Average no. links per member	Network type and parameters	Mean frequency of contact per link per day
Households without older adults (2,730)	1–2 adults, 0–4 children, 0–4 teenagers, mean size 3.13	2.13	Fully connected	6
Households with older adults (742)	1–2 older adults, mean size 1.75	0.75	Fully connected	6
Extended families or neighborhoods (800)	0–2 older adults, 0–8 adults, 0–8 teenagers, 0–8 children, mean size 12.5	11.5	Fully connected	1
Child classes (69)	1 class per child, 20–35 children in each	4	Ring network, 2 neighbors on either side	6
Child random (1)	All children	3	Random network link density 3/1,769	1
Teenager classes (264)	6 classes per teenager, 20–35 teenagers in each	4	Ring network, 2 neighbors on either side	1
Teenager random (1)	All teenagers	3	Random network link density of 3/1,129	1
Adult work (351)	1 work group per adult, 10–50 adults in each	6	Ring network, 3 neighbors on either side	1
Adult random (1)	All adults	3	Random network link density of 3/5,849	1
Older adult gathering (156)	1 gathering per person, 5–20 persons in each	4	Ring network, 2 neighbors on either side	1
Older adult random (1)	All older adults	3	Random network link density of 3/1,249	1
Overall random (1)	All age classes	25	Random network link density of 25/9,999	1/25 a day

All children and teenagers attended preschool or school; children attended 1 class/day, while teenagers attended 6 (classes of 20 to 35 children or teenagers). All adults went to work daily, where they interacted with other adults (work group size 10–50), and all older adults attended gatherings with other older adults (gathering size 5–20). For links within school classes, work, and gatherings of older adults, we assumed the simplest subnetwork that imposes local clustering: a ring lattice in which a person is linked to 2 (for children or teenager classes and gatherings of older adults) or 3 (adult work) neighboring persons on each side along the ring. Mean link contact frequencies for children in a class are 6/day. Teenager classes, adult work, and gatherings of older adults have mean link contact frequencies of 1/day.

To represent additional within-age class interactions, such as extracurricular activities, playgrounds, bowling leagues, or friends, persons are randomly linked to an average of 3 other persons of the same age class (mean link contact frequency 1/day). Finally, to emulate a somewhat patterned set of random contacts from commercial transactions and other ventures into public spaces, we impose a random overall network across all age classes with a mean of 25 links/person to yield 1 contact/person/day (mean link contact frequency 0.04/day).

## Behavioral Rules

**Figure 2:**
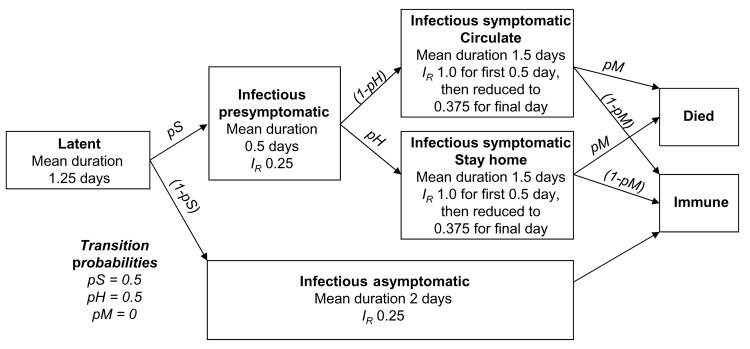
Natural history of influenza in our model. Duration of each state for a given person is chosen from an exponential distribution. State relative infectivity (I_R_) and mean state duration were chosen to reflect the infectivity variation of Ferguson et al. (*10**,**11*) (see Figure 3). Transition probabilities between presymptomatic and postsymptomatic states are also noted. For symptomatic persons who stay at home, link frequencies outside the household are reduced by 90%.

The spread of influenza within the contact network is modeled as a series of 2 classes of events: transition of a person between disease states and person-to-person transmission of influenza. Disease state transitions follow the natural history of influenza (Figure 2). After the latent state, an infected person transitions to an infectious presymptomatic state or an infectious asymptomatic state with probability pS or 1 – pS, respectively. Those with symptoms either stay home with probability pH, thus influencing their contacts, or continue to circulate with probability 1 – pH. Infected asymptomatic persons continue interacting without behavioral changes. Persons who are symptomatic die or become immune with probability pM or 1 – pM, respectively, and asymptomatic persons become immune. Because this final transition does not influence the spread of the disease, we use pM = 0.

Person-to-person transmission events are evaluated at the beginning of each period during which a person is infectious. Assuming contact events are statistically independent, a transmission time for each infectious person's links within the contact network is chosen from an exponential distribution with a mean of the link's contact frequency scaled by I_D_ × I_R_ × I_A_ × S_P_ × S_A_, where I_D_ is the infectivity of the disease, I_R_ is the relative infectivity of the disease state, S_P_ is the susceptibility of people to the disease (here taken as 1.0), I_A_ is the relative infectivity of the person who is transmitting, and S_A_ is the relative susceptibility of the person receiving. If the transmission time is less than the period during which the person will be in an infectious state (also chosen from an exponential distribution with the prescribed means; Figure 2), transmission is scheduled at the chosen time. Otherwise, transmission along that link does not occur during that period. All transmission parameters and contact frequencies may be modified in each of the states, as well as varied among age classes by relative scaling factors such as I_R_. In this way, disease representations and mitigation strategies are implemented.

**Figure 3:**
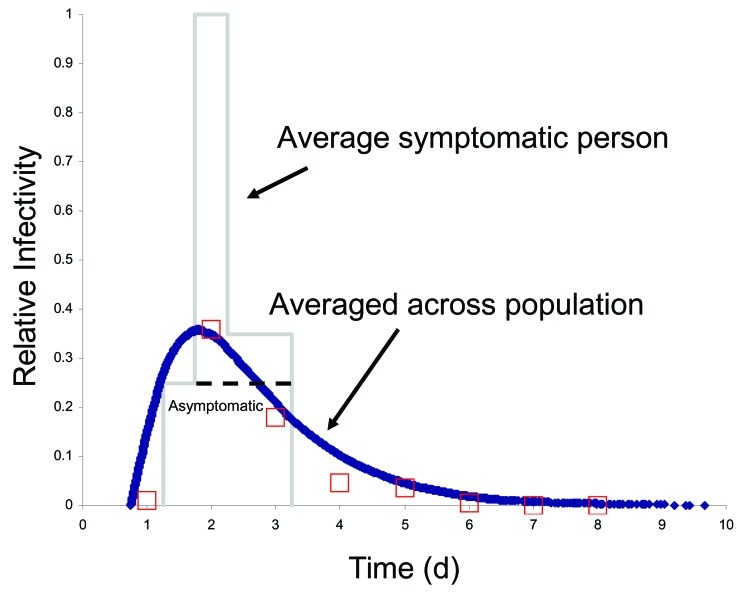
Functional behavior of I_R_ with time. Although infectivity of an asymptomatic person is constant with time (I_R_ 0.25), infectivity of a symptomatic person changes from infectious presymptomatic (I_R_ 0.25) to early infectious symptomatic (I_R_ 1.0) to late symptomatic (I_R_ 0.375). A symptomatic person with mean state periods as denoted in Figure 2 is shown in gray (asymptomatic with dashed line). Because state periods are different for each person (given by exponential distributions) and half of the infected persons are asymptomatic, the average population scale I_R_ in time is smoothed as shown in blue. Both disease state periods and I_R_ values were chosen to honor the clinically derived natural history of influenza (*12**–**14*), selected viral shedding data shown as open red squares (*15*), and the model of Ferguson et al. (*10**,**11*).

Most influenza-specific parameters used here reflect those of (*10**,**11*). We approximated normal influenza viral shedding data (*15*) with a time varying infectivity through choice of state periods and relative infectivity scaling factors (Figure 2 and Figure 3). The latent period is a constant (0.75 days) followed by a variable period (mean 0.5 days). The presymptomatic period (mean 0.5 days) has an I_R_ of 0.25 after which it increased to 1.0 for the first part of the symptomatic period (mean 0.5 days), when viral shedding is maximum and coughing begins. I_R_ is then reduced to 0.375 for the remainder of the infectious symptomatic period (mean 1 day). For infectious asymptomatic persons, I_R_ was set at 0.25 for a mean period of 2.0 days, making these persons half as infective as those with symptoms. We chose pS as 0.5, pH as 0.5 for adults and older adults and pH as 0.9 for children and teenagers. When a person is in the symptomatic stay-home state, we reduce the frequency of all nonhousehold connections by 90%. Because children and teenagers have closer contact with others and are less likely to wash hands or control coughs (*16*), they are more infective and susceptible: I_A_ and S_A_ are both 1.5 for children, 1.25 for teenagers, and 1.0 for adults and older adults. Finally, I_D_ is adjusted to yield specified attack rates within the community.

## Results

We first show the spread of influenza within our unmitigated base case defined with parameters specified above and with I_D_ chosen to yield an infected attack rate ≈50% to reflect the 1957–58 Asian influenza pandemic (*10*). Unless otherwise noted, we report infected attack rates and refer to them as simply attack rates rather than reporting the illness attack rate which is half of this value (pS = 0.5). We then demonstrate the design of effective local mitigation strategies for the base case that focus on targeted social distancing. Finally, we extend these results to design strategies for more infectious strains and for changes to the underlying infectious contact network that deemphasize the role of children and teenagers.

All simulations are initialized by infecting 10 randomly chosen adults with the assumption that adults are first to be infected through business travel or interaction with visitors from outside the community (*5*). Some of these initial infections instigate others and grow into an epidemic. Results vary across multiple realizations of the community network and random choice of initially infected adults (controlled by random number seed) not all of which yield an epidemic, defined when the number infected is >1% of the population. For every set of parameters, we conducted >100 simulations with different random number seeds and collected statistics for all simulations and for only those that result in epidemics (Table 2).

**Table 2:** Results for base case and miigation strategies*

Strategy	Averages for all simulations																		Averages for simulations with epidemics																	
	No. simulations		Total infected					Total time (d)				Peak infected		Time to peak (d)					No. epidemics		Total infected					Total time (d)				Peak infected				Time to peak (d)		
Case 1: Base case pandemic influenza																																					
Average		1,000			4,908			81			688				35			978				5,018			82				703				36			
SD					748			14			121				8							153			11				66				6			
Case 2: Schools closed after 10 symptomatic cases, compliance 90%																																					
Average	100			3,877				113			326				48			99				3,916			114				329					48			
SD				468				22			64				13							259			19				56					12			
% reduction from base case				21				-40			53				-36							22			-39				53					-34			
Case 3: Schools closed after 10 symptomatic cases, nonschool contacts doubled, compliance 90%																																					
Average	100			5,604			76			850				34			95				5,898			79				894				35			
SD				1,293			18			206				9							122			10				72				6			
% reduction from base case				-14			6			-24				4							-18			4				-27				2			
Case 4: Schools closed after 10 symptomatic cases, children and teenagers kept home, household contacts doubled, compliance 90%																																					
Average	100			341				60			43				16			93				361			62				45					17			
SD				209				25			20				12							203			24				19					12			
% reduction from base case				93				26			94				53							93			25				94					52			
Case 5: Schools closed after 10 symptomatic cases, children and teenagers kept home, household contacts doubled, compliance 50%																																					
Average	100			1,551				135			90				47			95				1,630			141				94					49			
SD				692				49			40				31							614			42				37					30			
% reduction from base case				68				-67			87				-33							68			-72				87					-36			
Case 6: Schools closed after 10 symptomatic cases, children kept home, household contacts doubled, compliance 90%																																					
Average	100			2,539				116			199				49			96				2,642			120				206					51			
SD				661				30			66				17							433			23				56					14			
% reduction from base case				48				-44			71				-38							47			-46				71					-40			
Case 7: All with symptomatic cases stay at home, compliance 90%																																					
Average		100				3,692			91				408			41				94			3,926				95				433				43	
SD						1,031			25				130			14							458				17				85				10	
% reduction from base case						25			-12				41			-16							22				-16				38				-20	

*Cases 2–7 are targeted social distancing strategies. Negative percent reductions reflect percent increases. Epidemics are defined as >100 infected. SD, standard deviation.

### Unmitigated Base Case

**Figure 4:**
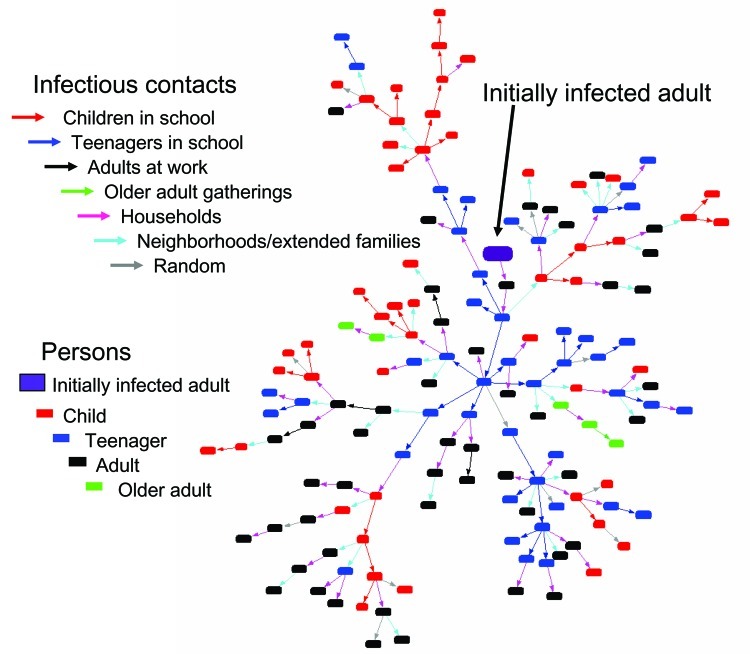
Initial growth of an infectious contact network. Colored rectangles denote persons of designated age class, and colored arrows denote groups within which the infectious transmission takes place. In this example, from the adult initial seed (large purple rectangle), 2 household contacts (light purple arrows) bring influenza to the middle or high school (blue arrows) where it spreads to other teenagers. Teenagers then spread influenza to children in households who spread it to other children in the elementary schools. Children and teenagers form the backbone of the infectious contact network and are critical to its spread; infectious transmissions occur mostly in the household, neighborhood, and schools.

**Figure 5:**
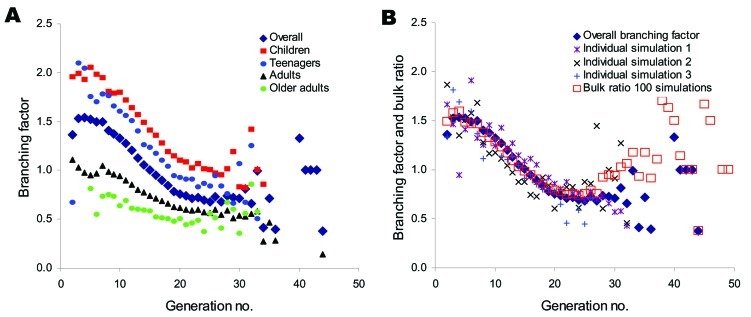
Branching factor and the approximation of the reproductive number *R_o_*. A) Overall and age class–specific branching factors as a function of generation averaged over 100 simulations. The standard deviations of these averages can be large (<0.72 at the peak value for teenagers) and reflect the heterogeneity within the person contact networks and from community to community. B) Branching factors for overall average and 3 example simulations compared with the bulk ratio of infections in a generation to those in the previous generation pooled across 100 simulations. We chose the maximum value of the bulk ratio (1.6) as an approximation of the reproductive number *R_o_*.

The sequence of infected persons can be represented as an expanding network of infectious transmissions (Figure 4). The number of secondary infections produced by an infected person, or branching factor, is easily visualized within the infectious contact network. The average branching factor depends on the person's age class and generation during the epidemic (Figure 5A). The maximum value within the first 10 generations is 2.05 (standard deviation [SD] 0.57) for children, 2.09 (SD 0.72) for teenagers, 1.11 (SD 0.43) for adults, 0.81 (SD 0.47) for older adults, and 1.54 (SD 0.36) for the entire population. Variability (large SD, especially for specific age classes) reflects the heterogeneity inherent within community contact networks of this size (Figure 5B).

The backbone of infectious contact networks is formed primarily of children and teenagers with infectious transmissions mostly in the household, neighborhood, and schools. Infectious transmissions are highest in households without older adults (39%, SD 3%), followed by extended families or neighborhoods (25%, SD 1%), schools (19%, SD 1%), work (7%, SD 2%), combined random groups (9%, SD 2%), and households with older adults (1%, SD 0.1%). On average, 78% (SD 2%) of children and 71% (SD 3%) of teenagers become infected. Adults (attack rate 44% of adults, SD 2%) get influenza mainly from children, teenagers, and other adults within the family. Older adults, who contact children and teenagers only through the extended family or neighborhoods and the random overall network, are relatively isolated (attack rate 23% of older adults, SD 2%).

Children and teenagers compose only 29% of the population yet they are responsible for 59% (SD 4.5%) of infectious contacts, adults for 38% (SD 7.9%), and older adults for 3% (SD 0.6%) (Table 3). Approximately half of infectious contacts of either children or teenagers are within the same age class (19%, SD 0.8% and 9%, SD 0.7%, respectively). Adults get influenza from children or teenagers at approximately the same frequency (24%, SD 1.6%) as from other adults (26%, SD 5.9%). Older adults are equally likely to get influenza from children or teenagers as from adults or older adults (2%, SD 0.3%). Transmission to children or teenagers from adults is 10% (SD 1.8%) and nearly none by older adults. These transmission results are supported by recent field studies that show children who go to preschool or school are more likely to contact influenza and their family members are also more likely to become ill (*17**,**18*) as well as that a person is also more likely to be infected when exposed to children or teenagers than to adults (*14*).

**Table 3:** Unmitigated base case infectious contact fractions (% of the total no. of infectious contacts) between age classes*

Class	To children	SD	To teenagers	SD	To adults	SD	To older adults	SD	Total	SD
From children	18.6	0.8	2.9	0.3	16.1	1.1	1.2	0.2	38.8	2.4
From teenagers	2.4	0.8	9.1	0.7	8.0	0.5	0.6	0.1	20.1	2.1
From adults	6.0	0.6	3.8	1.2	26.0	5.9	2.1	0.4	38.0	7.9
From older adults	0.2	0.1	0.2	0.1	0.9	0.9	1.8	0.3	3.1	0.6
Total	27.3	2.2	16.0	2.2	50.9	7.7	5.8	0.9		

*SD, standard deviation.

**Figure 6:**
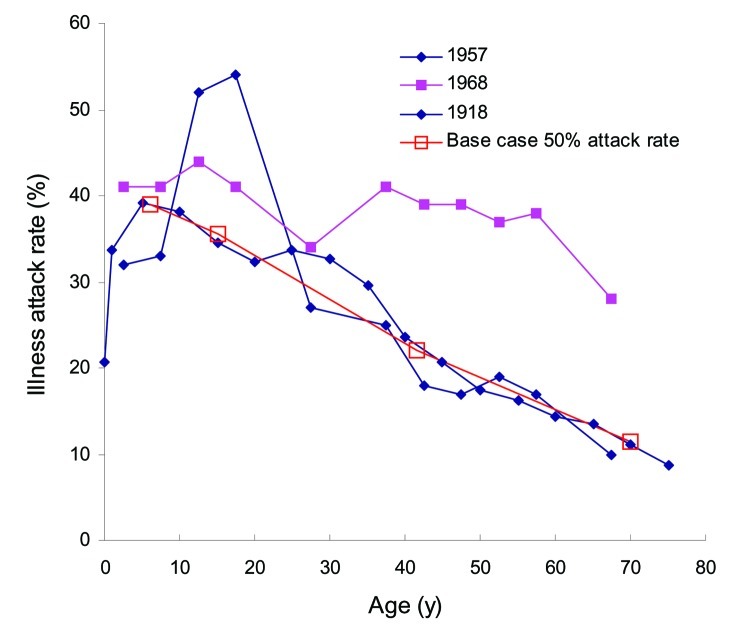
Comparison of simulated age class–specific illness attack rates with past pandemics. Simulated illness attack rates (half the infectious attack rate) for the unmitigated base case are close to those found in studies of historic pandemics in 1957 (*19*), 1968 (*20*), and 1918 (*21*). Notable differences are the 1968 Hong Kong flu, which had less effect on youth and 1957–58 Asian flu, which had greater effect; however, historic data are inherently uncertain. Closer correspondence to either of these 2 cases could be achieved through changes in I_A_ or S_A_ or modification of the underlying social contact network (see Results) because the network was likely different from that of a small town of today.

Reasonable correspondence is observed (Figure 6) between age class–specific attack rates and those of past pandemics (*19**–**21*). Infections transmitted within each environment are also consistent with other simulation studies (*10**–**14*). The maximum value of the overall branching factor (Figure 5) reflects the often-cited reproductive number R_o_. However, how R_o_ should be calculated from small-community data such as ours is ambiguous (*10**,**11**,**14*). To estimate R_o_, we pooled results across 100 communities (simulations) to reflect a population of 1 million (Figure 5B). The maximum value of the bulk ratio (new infections to old) within the first 10 generations is 1.6, and we choose it as our estimate of R_o_. An R_o_ of 1.6 with an attack rate of 50% matches recent pandemic simulation results (*10**,**14*) and lies within the range (1.5–1.7) for the 1957–58 influenza pandemic (Figure 5B) (*10*).

### Base Case–Targeted Social Distancing

High infectiousness and a high number of contacts, many like-to-like, create a zone of high infectious contact centered on children and teenagers within the community's social network. Targeting this zone can protect the community at large.

First, we examined closing schools. Although contacts in classes are removed, those in all other groups may increase because children and teenagers spend more time at home, in neighborhoods, with friends, and in public spaces. We assume that school closure at a minimum doubles household contacts. Closing schools with 90% compliance the day after 10 symptomatic cases reduces the attack rate to 22% (Table 2). However, if we assume that school closure doubles all link contact frequencies for children or teenagers within their nonschool groups, attack rates are increased by 18% (Table 2).

**Figure 7:**
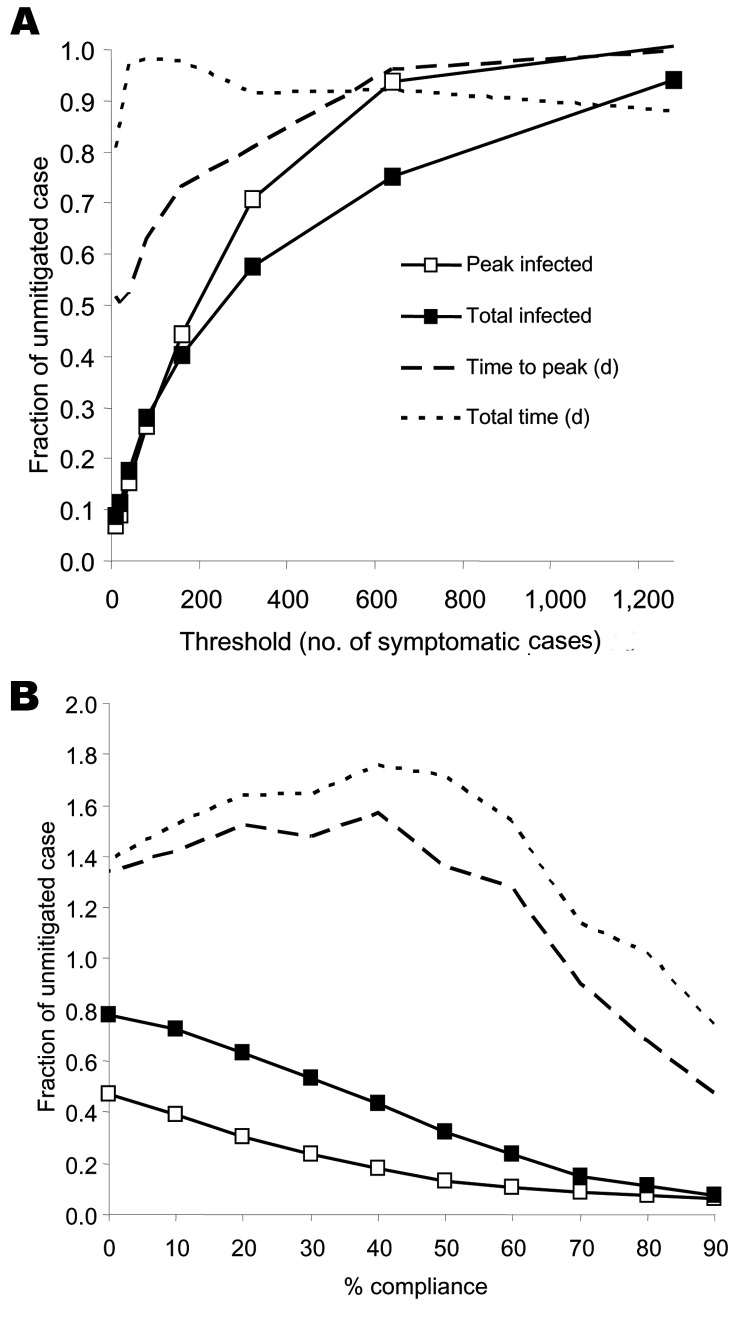
Fraction of unmitigated base case attack rate for targeted social distancing of children and teenagers as a function of A) implementation policy threshold given by the number of symptomatic cases (compliance at 90%) and B) compliance with staying at home (implementation policy threshold at 10 symptomatic cases, 0% compliance closes schools alone). Each point represents the average of simulations of 100 that yielded epidemics (>100 infected). Standard deviations for variation of threshold are <3% of the total population. However, for compliance variation, standard deviations increase to a maximum of 7% of the total population at a compliance of 30%.

Alternatively, we send all children and teenagers home after school closure to remain for the duration of the pandemic. Now contact frequencies are reduced by 90% for all groups that contain only children or teenagers (classes and their random networks) and doubled, as before, for children or teenagers in households. In the extended family or neighborhood and the random overall networks, child or teenager contact frequencies are also reduced by 90%. Thus, although children and teenagers are restricted to the home, adults and older adults go about their day-to-day routines, except that they avoid children or teenagers who are not household members. Imposing this strategy the day after 10 symptomatic cases reduces attack rates by 93% (Table 2). Waiting until 80 symptomatic cases reduces attack rates by 73% (Figure 7A).

To evaluate the tradeoff between effectiveness and public compliance, we reduced the percentage of nonschool and nonhousehold contacts that have their frequencies reduced with the child and teenager stay-at-home policy (Figure 7B). At 50% compliance, attack rates can be reduced by 68% (Table 2). Reduction in compliance also increases the time scales for the epidemic. Epidemics lengthen above the base case and reach a factor of ≈1.8 at 40% compliance (Figure 7B).

Other social distancing strategies can be considered. Because children outnumber teenagers and children are more infective and susceptible, what happens if only children are distanced, while teenagers attend school and follow their usual routines? At 90% compliance, this strategy reduces the attack rates by 47% (Table 2). What if all sick persons remain at home when symptomatic? At 90% compliance this strategy reduces attack rates by 20% (<25% of infectious persons are influenced as pS × pH = 0.25 for adults only) (Table 2).

### More Infective Strains and Contact Networks with Less Emphasis on the Young

We have modeled an influenza strain with an infectivity representative of the 1957–58 Asian influenza pandemic and a social contact network reflective of a stylized US town. Although results for the unmitigated base case yield age class–specific attack rates similar to those for past epidemics (Figure 6), will the targeted social distancing strategies found above remain effective if 1) the strain is more infective or 2) the importance of the young is deemphasized?

To explore these questions, we considered 3 increases in disease infectivity I_D_ by factors of 1.25 (attack rate ≈66%, R_o_ ≈ 1.8), 1.5 (attack rate ≈75%, R_o_ ≈ 2.0), and 2.0 (attack rate ≈86%, R_o_ ≈2.4). These increases encompass and exceed the 1918–19 Spanish influenza pandemic (R_o_ 1.8–2.0) (10). We also sequentially removed enhanced transmission by children and teenagers and thus the zone of high infectious contact that we have designed social distancing strategies to target. We created 3 variations: the first removed relative infectivity and susceptibility enhancement of children and teenagers (I_A_ and S_A_ 1.0) (variation 1); the second increased frequency of contact within the work environment by a factor of 4 to give adults the same number of contacts as younger persons (variation 2); and the third combined variations 1 and 2. For each of the resulting set of 4 cases (base, variation 1, variation 2, and variation 1 and 2), I_D_ was altered slightly to maintain the reference of ≈50% infected attack rate for R_o_ ≈1.6.

**Figure 8:**
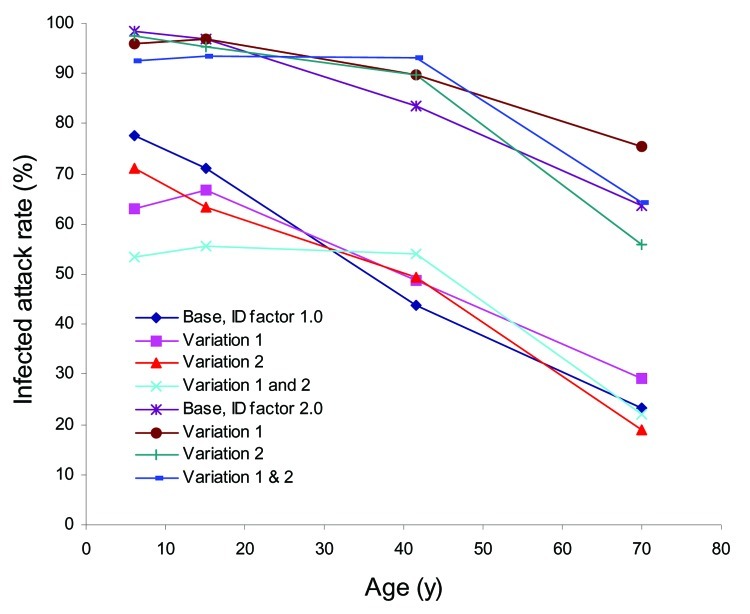
Unmitigated age-specific attack rate results for disease infectivity (I_D_) factors of 1.0 and 2.0 and base case, variation 1 (removal of relative infectivity and susceptibility), variation 2 (increase in work group frequency of contact to give all children, teenagers, and adults the same overall contact frequencies), and variations 1 and 2 combined. Illness attack rates shown in Figure 6 are half these values.

As I_D_ increases, age specific–attack rates increase (Table 4). As we remove differences in the number of contacts and/or relative infectivity and susceptibility (I_A_, S_A_) between young and adults, the infected attack rates systematically shift from young persons to adults (Figure 8). These results suggest that for such situations, social distancing strategies must be devised that focus on more than children and teenagers alone.

**Table 4:** Unmitigated case results for *R_o_* and average attack rates (%) for increasing *I_D_* and base case, variation 1, variation 2, and variations 1 and 2 combined*

Type	*I_D_* factor	*R_o_*	Attack rates									
			Overall	SD	Children	SD	Teenagers	SD	Adults	SD	Older adults	SD
Base case	1.0	1.6	51	1.7	79	2.3	72	2.7	45	1.8	23	2.0
	1.25	1.8	66	1.2	90	1.1	85	1.7	61	1.5	36	2.1
	1.5	2.0	75	0.7	95	0.6	92	1.1	71	0.9	47	1.9
	2.0	2.4	86	0.6	99	0.3	97	0.5	84	0.7	64	2.0
Variation 1	1.0	1.5	52	2.2	65	2.8	68	3.2	50	2.2	30	2.5
	1.25	1.7	70	1.3	82	1.4	84	1.8	68	1.5	47	2.3
	1.5	1.9	80	0.8	90	1.0	91	0.9	79	0.9	60	2.0
	2.0	2.4	90	0.5	96	0.6	97	0.7	90	0.6	76	1.6
Variation 2	1.0	1.5	52	1.9	72	2.6	64	2.9	50	2.1	19	1.7
	1.25	1.8	68	1.0	87	1.3	81	1.6	68	1.3	31	2.1
	1.5	1.9	78	0.8	93	0.9	89	1.2	79	1.0	41	2.2
	2.0	2.3	88	0.5	98	0.4	96	0.7	90	0.7	57	1.9
Variations 1 and 2 combined	1.0	1.5	52	2.0	55	2.3	57	2.7	56	2.3	23	1.7
	1.25	1.8	70	1.1	74	1.8	76	1.9	75	1.2	37	2.0
	1.5	2.0	80	0.8	84	1.2	85	1.2	85	0.8	48	2.0
	2.0	2.4	90	0.4	93	0.6	94	0.9	94	0.5	65	1.8

*Variation 1 is removal of relative infectivity and susceptibility; variation 2 is an increase in work group frequency of contact to give all children, teenagers, and adults the same overall contact frequencies. Average attack rates accumulate over only those simulations that resulted in epidemics (>100 infected). *R_O_*, reproductive number; *I_D_*, disease infectivity; SD, standard deviation.

To find effective targeted social distancing strategy combinations across the range of disease infectivity and infectious contact networks, we formulated 5 strategies and applied them individually and in combination: 1) school closure (S) where the contact frequency within schools was reduced 90% and children and teenager's household contacts were doubled; 2) children and teenagers social distancing (CTsd) where their contact frequencies in all nonhousehold and nonschool groups were reduced 90% and their household contacts doubled; 3) adult and older adult social distancing (AOAsd) where their contact frequencies in all nonhousehold and nonwork groups were reduced 90% and household contacts doubled; 4) liberal leave (LL), where all children and teenagers and 90% of adults withdraw to the home when symptomatic; and 5) work social distancing (Wsd) where the contact frequency within work groups was reduced 50%. For each combination, we implemented the strategy(ies) the day after 10 symptomatic cases and conducted 100 simulations.

As I_D_ increases, more strategies must be combined to keep the attack rate <10% (Table 5, shaded values). As children and teenagers become less prominent, targeting adults becomes important, even at an I_D_ factor of 1. For an I_D_ factor of 1.5 (as infective as the 1918-19 Spanish influenza pandemic) and across all variations, both the young and adults must be targeted and all strategies must be implemented to effectively mitigate the epidemic. However, for an I_D_ factor of 2.0, we can at best reduce the attack rate to 20 –40% through full strategy combination, not ideal but still a significant benefit.

**Table 5:** Mitigated case average attack rates (%) for increasing *I_D_* and base case, variation 1, variation 2, and variations 1 and 2 combined*

No.	Strategy combination					Base case *I_D_* factor				Variation 2 *I_D_* factor				Variation 1 *I_D_* factor				Variations 1 and 2 *I_D_* factor			
	S	CTsd	AOAsd	LL	Wsd	1	1.25	1.5	2	1	1.25	1.5	2	1	1.25	1.5	2	1	1.25	1.5	2
1						51	66	75	86	52	68	78	88	52	70	80	90	52	70	80	90
2					Wsd	48	63	72	84	41	60	71	83	47	66	77	88	35	58	72	86
3				LL		41	57	67	79	37	57	68	82	36	57	70	84	28	55	69	84
4				LL	Wsd	39	55	65	78	30	49	62	77	30	53	67	82	12	42	60	78
5			AOAsd			38	51	59	70	40	58	68	79	25	46	58	72	33	56	69	80
6			AOAsd		Wsd	35	48	56	66	30	47	58	71	18	39	51	66	9	37	53	71
7			AOAsd	LL		32	46	55	66	28	48	60	73	13	36	50	67	11	40	57	74
8			AOAsd	LL	Wsd	30	43	52	63	21	40	51	66	10	32	46	62	4	23	42	64
9		CTsd				41	58	69	82	45	64	75	86	41	63	76	88	46	67	78	88
10		CTsd			Wsd	37	55	66	79	31	53	66	80	32	57	71	85	21	52	67	83
11		CTsd		LL		29	48	60	75	26	50	64	78	20	47	63	80	19	49	65	81
12		CTsd		LL	Wsd	27	45	57	72	16	40	55	72	14	41	58	77	6	32	53	74
13		CTsd	AOAsd			29	46	56	68	34	55	66	78	15	40	54	70	27	54	67	79
14		CTsd	AOAsd		Wsd	26	42	52	64	20	41	54	69	9	31	45	63	5	30	50	69
15		CTsd	AOAsd	LL		22	39	51	64	18	42	56	72	7	29	45	64	7	35	55	73
16		CTsd	AOAsd	LL	Wsd	20	37	48	61	10	32	47	63	5	22	39	58	3	16	37	61
17	S					41	61	73	85	45	66	77	87	47	68	79	90	51	69	80	90
18	S				Wsd	36	57	70	83	30	54	68	83	38	62	75	88	29	56	71	85
19	S			LL		23	47	62	78	23	49	65	80	20	50	66	83	22	51	67	83
20	S			LL	Wsd	19	44	59	76	9	38	55	74	13	44	62	80	6	35	55	76
21	S		AOAsd			26	47	59	74	34	56	69	81	16	44	60	76	34	58	70	82
22	S		AOAsd		Wsd	20	41	55	70	14	41	57	73	8	35	52	71	7	36	55	74
23	S		AOAsd	LL		11	35	51	68	12	40	57	74	5	28	48	69	8	38	57	75
24	S		AOAsd	LL	Wsd	9	32	47	65	5	27	45	66	4	20	41	64	3	14	39	65
25	S	CTsd				4	26	50	73	15	47	64	80	12	46	64	82	34	58	71	84
26	S	CTsd			Wsd	3	15	40	68	3	21	46	71	5	32	55	78	6	36	56	77
27	S	CTsd		LL		2	7	29	60	3	21	45	70	3	17	43	70	5	33	54	75
28	S	CTsd		LL	Wsd	2	6	20	54	2	6	24	57	2	9	31	64	2	9	33	64
29	S	CTsd	AOAsd			2	4	13	44	4	24	48	70	2	4	15	49	8	37	56	73
30	S	CTsd	AOAsd		Wsd	2	3	7	30	2	5	16	49	2	3	6	28	2	5	20	54
31	S	CTsd	AOAsd	LL		2	3	9	34	2	7	27	58	2	3	7	36	3	11	35	63
32	S	CTsd	AOAsd	LL	Wsd	2	3	6	25	2	3	8	37	2	2	5	20	2	3	9	39

*Variation 2 is an increase in work group frequency of contact to give all children, teenagers, and adults the same overall contact frequencies; variation 1 is removal of relative infectivity and susceptibility. *I_D_*, disease infectivity; S, school closure; CTsd, children and teenagers social distancing; AOAsd, adults and older adults social distancing; LL, liberal leave; Wsd, work social distancing. Shaded numbers denote strategy combinations that reduce the attack rate to <10% of the population (illness attack rate <5%). Average attack rates accumulate over only those simulations that resulted in epidemics (>100 infected). Average standard deviation across the entire set of simulations was 2.2% with a maximum of 7.6%.

## Discussion

Results for our stylized small town suggest that targeted social distancing strategies can be designed to effectively mitigate the local progression of pandemic influenza without the use of vaccine or antiviral drugs. For an infectivity similar to that of the 1957–58 Asian influenza pandemic, targeting children and teenagers, by not only closing schools but also by keeping these age classes at home, was effective. However, given uncertainty in the infectivity of the influenza strain, underlying social contact network, or relative infectivity/susceptibility of the young versus adults, planning to implement strategies that also target adults and the work environment is prudent. To mitigate a strain with infectivity similar to that of the 1918–19 Spanish influenza pandemic, simulations suggest that all young and adults must be targeted regardless of the likely enhanced transmission by the young.

Implementation of social distancing strategies is challenging. They likely must be imposed for the duration of the local epidemic and possibly until a strain-specific vaccine is developed and distributed. If compliance with the strategy is high over this period, an epidemic within a community can be averted. However, if neighboring communities do not also use these interventions, infected neighbors will continue to introduce influenza and prolong the local epidemic, albeit at a depressed level more easily accommodated by healthcare systems.

Our design approach explicitly implements disease-host interaction within the social contact network where the disease spreads. Measuring contact networks within communities for the spread of infectious diseases requires focused research that combines sociology, public health, and epidemiology. Such networks will likely differ across cultures, between urban and rural communities, and with community size. With the aid of detailed demographic data, expert elicitation of social scientists and community members, behavioral surveys, and possibly experiments, a network could be constructed for any community of interest. Configurations that consider, for example, college campuses or military reservations may be of use given that the highest death rate of any group in the 1918–19 Spanish influenza pandemic was in young adults (*22*).
